# Evaluation of the depiction ability of the microanatomy of the temporal bone in quarter-detector CT: Model-based iterative reconstruction vs hybrid iterative reconstruction

**DOI:** 10.1097/MD.0000000000015991

**Published:** 2019-06-14

**Authors:** Ryo Kurokawa, Eriko Maeda, Harushi Mori, Shiori Amemiya, Jiro Sato, Kenji Ino, Rumiko Torigoe, Osamu Abe

**Affiliations:** a Department of Radiology, Graduate School of Medicine, University of Tokyo; b Department of Radiation Technology, University of Tokyo Hospital; c Canon Medical Systems Corporation, Japan.

**Keywords:** hybrid iterative reconstruction, model-based iterative reconstruction, quarter-detector CT

## Abstract

Little is known regarding differences between model-based iterative reconstruction (MBIR) and hybrid iterative reconstruction (HIR) in temporal bone computed tomography (CT). This study compared the ability to depict microstructures in temporal bone in quarter-detector CT (QDCT) between MBIR and HIR.

Sixty-two temporal bones in 31 consecutive adult patients who underwent QDCT were included. Reconstruction was performed with Forward projected model-based Iterative Reconstruction SoluTion (FIRST) BONE mild mode and Adaptive Iterative Dose Reduction 3D (AIDR3D) enhanced mild mode. Imaging quality was graded for 3 microstructures (spiral osseous lamina, tympanic membrane, and singular canal).

Spiral osseous lamina was significantly well-delineated in the AIDR3D enhanced group, compared with the FIRST group. In nearly all cases with FIRST, spiral osseous lamina was poorly defined. Although there was no significant difference, depiction of the tympanic membrane and singular canal tended to be better with AIDR3D enhanced mode.

Routine reconstruction for preoperative temporal bone CT should be performed with HIR, rather than MBIR.

## 1 Introduction

Quarter-detector computed tomography (QDCT) has a detector of 0.25 × 0.25 mm and 2-fold greater spatial resolution in the in-plane/body-axis direction, compared with conventional multi detector-row CT. Thus, it is possible to obtain detailed biometric information that could not be detected in the conventional CT. Three different scan modes with different numbers of channels and reconfigurable matrices are available. Among these, super high-resolution (SHR) mode enables reconstruction of up to a 2048 × 2048 matrix with slice thickness of 0.25 and slice spacing of 0.25 mm.

Forward projected model-based Iterative Reconstruction SoluTion (FIRST) is a commercialized model-based iterative reconstruction (MBIR) method available in QDCT. FIRST is a raw data-based 3D image reconstruction method that is considered to provide images with more reduced noise while preserving better spatial resolution than the hybrid iterative reconstruction (HIR) mode. Previous studies have reported that MBIR showed better imaging quality of many body parts compared with HIR.^[1–6]^ Few studies have reported inferiority of MBIR to HIR.^[7]^ Because of its recent introduction, little is known regarding differences between MBIR and HIR in temporal bone CT, even in QDCT. The present study aimed to compare the depiction ability for the microstructures in temporal bone in QDCT between MBIR (FIRST) and HIR (Adaptive Iterative Dose Reduction 3D (AIDR3D) enhanced).

## 2 Material and methods

This retrospective study was approved by the local ethics committee (IRB 2561-(16)), and the requirement for informed consent for study participation was waived.

### 2.1 Patients

Between May and July 2018, 62 temporal bones in 31 consecutive adult patients that underwent temporal bone CT with the SHR mode of QDCT (Aquilion Precision; Canon, Tochigi, Japan) were included. The mean age was 59 years (range, 24–86 years); the numbers of male and female patients were 15 and 16, respectively. Backgrounds of the patients were as follows: otitis media cholesteatoma (n = 8), chronic otitis media (n = 8), hearing loss without any mechanical problems (n = 5), ossicular chain disruption (n = 3), otosclerosis (n = 2), ear fullness without any mechanical problems (n = 2), ossicular malformation (n = 2), and temporal bone fracture (n = 1). The following 3 anatomical micro structures with different contrasts were selected for evaluation: spiral osseous lamina (soft tissue and bone in water), tympanic membrane (soft tissue in air), and singular canal (soft tissue in bone). Twenty-five tympanic membranes in 16 patients were excluded from the analysis because the structures had been surgically modified (number of tympanic membranes = 16) or modified by chronic otitis media or cholesteatoma (n = 9). All cases of spiral osseous laminae and singular canals were included in the analysis.

### 2.2 CT data acquisition

All patients underwent temporal CT via QDCT. Acquisition parameters were as follows: detector configuration, 160 × 0.25 mm; tube potential, 120 kV; tube current-time product was determined by auto exposure control. For all CT scans, FIRST BONE mild mode and AIDR3D enhanced mild mode were used for reconstruction in MBIR and HIR, respectively. Slice thickness was 0.25 mm, slice increment was 0.125 mm, axial matrix was 1024 × 1024 (smallest available matrix among the imaging methods reconstructible by FIRST), and region of interest (ROI) was 80 mm (centered in each ear).

### 2.3 Subjective image analysis

Subjective image quality was rated by two head and neck radiologists with 5 and 21 years of experience who were blinded to the details of the CT data sets; data were provided in a randomized order. A monitor with screen resolution 1200 × 1600 was used for evaluations. For CT viewing, CENTRICITY Radiology RA1000 (GE Healthcare, Milwaukee, WI) was used. Overall image quality was graded on a 3-point scale (1 = poor diagnostic image quality, 2 = moderate diagnostic image quality with limitations, 3 = excellent image quality). When readers’ scores differed, their average score was adopted as the final score. Window level and window width were set at 400 HU and 4000 HU, respectively.

### 2.4 Objective image analysis

Image noise (standard deviation of ROI measurement) was assessed by manually placed ROIs in the bone surrounding the vestibule, within the vestibule, and in air on axial images. For all measurements, the sizes, shapes, and positions of ROIs were held constant. The signal-to-noise ratio was calculated as the mean attenuation values in the ROI, divided by the image noise in the ROI.

### 2.5 Statistical analysis

Differences in scores between the 2 groups were analyzed using the Wilcoxon signed-rank test. The results were presented as means ± standard errors or means ± standard deviations. *P* < .05 was considered significant. Statistical analysis was performed with JMP (version 13.2.0; SAS Institute Inc., Cary, NC).

## 3 Results

**Figure 1:**
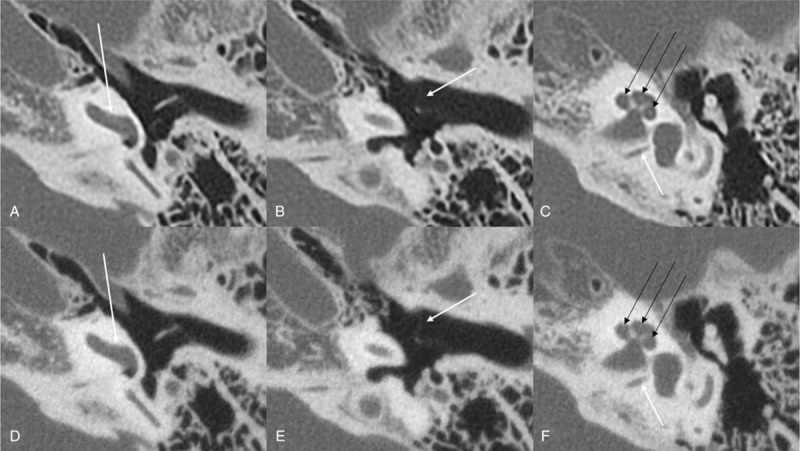
Temporal bone CT in a patient reconstructed with hybrid iterative reconstruction (HIR) (AIDR3D enhanced mild mode; A–C) and with model-based iterative reconstruction (MBIR) (Forward projected model-based Iterative Reconstruction SoluTion (FIRST) BONE mild mode; D–F). The spiral osseous lamina is clearly delineated in HIR (A; score 3), whereas it is nearly invisible in MBIR (D; score 1). The tympanic membrane is visible but blurred, both in HIR (B; score 2) and in MBIR (E; score 2). The singular canal is well-delineated both in HIR (white arrow in C; score 3) and in MBIR (F; score 3). Note that the spiral osseous lamina is also well-delineated in HIR (C; black arrows) and invisible in MBIR (F; black arrows). Two radiologists graded all 3 structures in an identical manner in this case.

**Figure 2:**
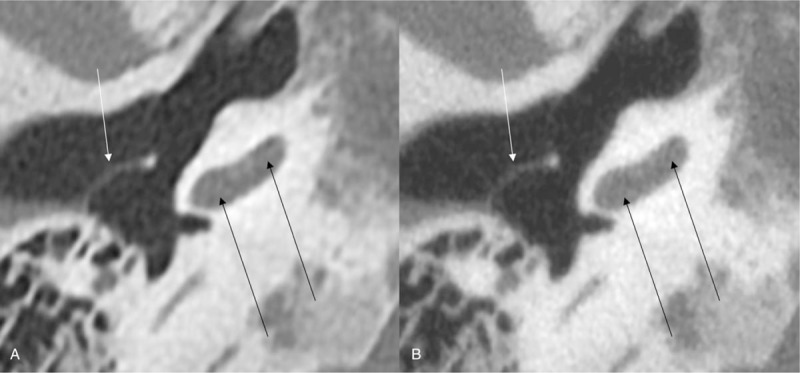
Temporal bone CT in a second patient reconstructed with hybrid iterative reconstruction (HIR) (A) and with model-based iterative reconstruction (MBIR) (B). The spiral osseous lamina is well-delineated in HIR (A; black arrows; score 3), but nearly invisible in MBIR (B; black arrows; score 1). The tympanic membrane is well-delineated both in HIR (A; white arrow; score 3) and MBIR (B; white arrow; score 1). Two radiologists graded both structures in an identical manner in this case.

The spiral osseous lamina was significantly well-delineated with AIDR3D enhanced mild mode, compared with FIRST BONE mild mode (Figs. 1 and 2). In almost all cases with FIRST (58/61 = 95.1%), the spiral osseous lamina was graded score 1. A tendency toward better depiction could be observed in the tympanic membrane and singular canal. The results of the scores of the three microstructures are summarized in Table 1.

**Table 1:**
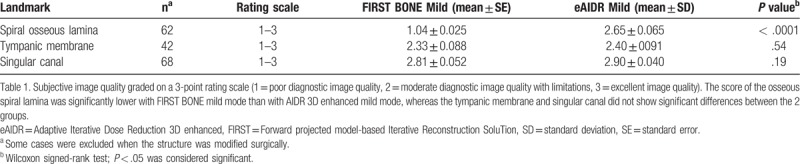
Summary of subjective image quality.

The CT value of bone was significantly higher with FIRST, while the CT value of air was significantly higher with AIDR3D enhanced. The image noises of bone and water were significantly higher in FIRST, while the image noise of air was significantly higher in AIDR3D enhanced. The radiation doses and image noises are summarized in Table 2 and Table 3, respectively.

**Table 2:**
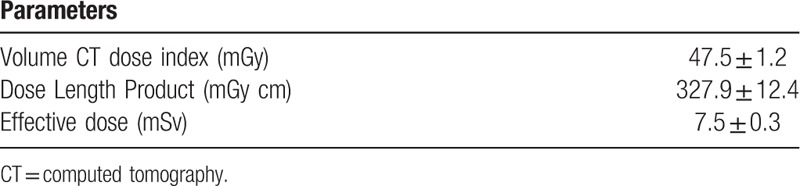
Radiation doses (average ± standard error).

**Table 3:**
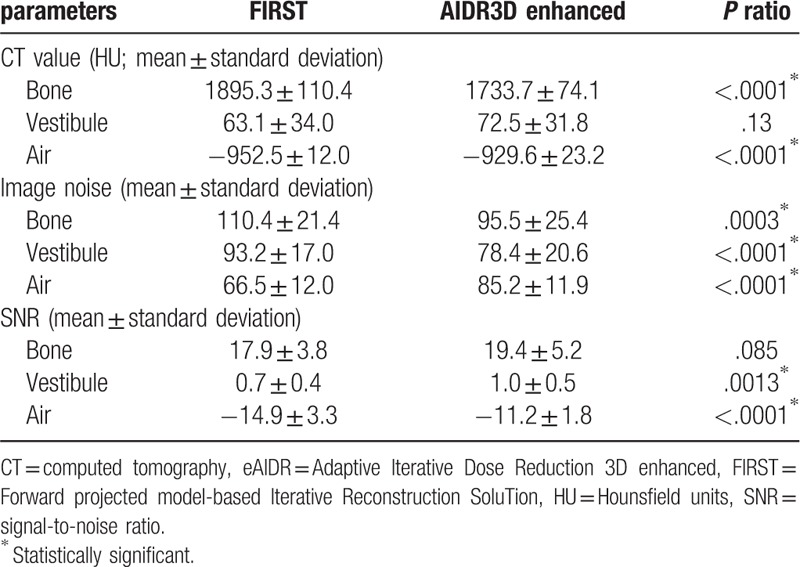
Quantitative assessment of image quality.

## 4 Discussion

There are few studies comparing the depiction ability of MBIR and HIR in QDCT. FIRST is a commercialized MBIR that jointly optimizes image quality in both sonogram and image spaces. By including both forward and statistical models in the projection data fidelity term, FIRST enables both high spatial resolution and reduced noise streaks. Images also undergo a regularization process optimized for specific organs (e.g., bone, heart, lung, and abdomen) to reduce image noise. Many studies have reported that MBIR showed superiority or non-inferiority in imaging quality or in radiation exposure reduction, compared with HIR in many other parts of the body^[1–6]^; few studies have reported the inferiority of MBIR, compared with HIR.^[7]^

In temporal bone CT, HIR is known for its superiority in spatial resolution and reduction of radiation exposure for FBP.^[8–10]^ However, there is little knowledge regarding the superiority of MBIR, compared with HIR, in temporal bone CT.

Because the CT value of bone was significantly higher in FIRST BONE mild mode, compared with AIDR3D enhanced mild mode, and there were no significant differences in the CT values of vestibule (perilymph) between the two modes, we expected that the spiral osseous lamina would be visualized more clearly in FIRST BONE mild mode. However, the results of the present study indicated that the spiral osseous lamina was significantly well-delineated with HIR (AIDR3D enhanced mild mode), compared with MBIR (FIRST BONE mild mode). The spinal osseous lamina and basilar membrane are coalescent structures that separate the outer lymphatic space of the cochlea into the vestibular floor and tympanic floor. Clear anatomy inside the cochlea is important in inner ear surgery, especially during electrode insertion in cochlear implantation.^[7,11]^ Therefore, routine reconstruction for preoperative temporal bone CT should be performed with HIR, rather than MBIR.

The following can be considered as potential sources for our results: FIRST BONE mode is optimized for high contrast tissues, although the spiral osseous lamina, combined with the basilar membrane, comprises bone and soft tissue in the lymphatic space; this generated a relatively low contrast among the 3 microstructures (spiral osseous lamina, tympanic membrane, and singular canal). Because of its lower contrast, spiral osseous lamina might have been eliminated by iteration; conversely, the relatively high-contrast tympanic membrane (soft tissue in air) and the singular canal (soft tissue in bone) were well-delineated in both reconstruction modes. The effects of other MBIR modes optimized for other organs, such as FIRST HEAD mode, low contrast detectability mode, or BODY mode, may improve the depiction of spiral osseous lamina; this awaits further investigation.

In conclusion, the depiction ability for the spiral osseous lamina in QDCT was significantly higher with HIR (AIDR3D enhanced mild mode), compared with MBIR (FIRST BONE mile mode). Although there was no significant difference, depiction of the tympanic membrane and singular canal tended to be better with HIR. Based on our study results, routine reconstruction for preoperative temporal bone CT should be performed with HIR, rather than MBIR.

## Author contributions

**Data curation:** Ryo Kurokawa, Eriko Maeda, Kenji Ino, Rumiko Torigoe.

**Methodology:** Ryo Kurokawa.

**Project administration:** Ryo Kurokawa.

**Resources:** Rumiko Torigoe.

**Supervision:** Eriko Maeda, Osamu Abe.

**Writing – original draft:** Ryo Kurokawa.

**Writing – review & editing:** Eriko Maeda, Harushi Mori, Shiori Amemiya, Jiro Sato, Osamu Abe.

Ryo Kurokawa orcid: 0000-0002-1186-8900.
